# Conservative Management of Class III Invasive Cervical Resorption Using the Heithersay Technique: A Case Report

**DOI:** 10.7759/cureus.106123

**Published:** 2026-03-30

**Authors:** Kalyana Ponangi

**Affiliations:** 1 Endodontics, Noble Community Clinics, Plover, USA

**Keywords:** endodontic therapy, glass ionomer cements, injuries, root resorption, tooth resorption

## Abstract

Invasive cervical resorption (ICR) is an uncommon condition involving the gradual loss of cervical tooth structure due to the invasion of fibrovascular tissue because of predisposing factors such as trauma, orthodontics, and intracoronal restorations. A 45-year-old female patient presented with a class III cervical lesion and a missing restoration on the maxillary left lateral incisor following trauma. Radiographic examination revealed a “moth-eaten” appearance and a negative pulp test when cold testing was performed. Since it was a Class III lesion, the Heithersay technique was the choice of treatment, which included the application of trichloroacetic acid (TCA 90%) as to induce coagulative necrosis. The lesion was then curetted, root canal treated, and restored with glass ionomer cement. One-year follow-up demonstrated satisfactory healing with no further resorption. The Heithersay approach proved to be successful in treating class III ICR lesions.

## Introduction

Invasive cervical resorption (ICR) is an uncommon clinical condition. It can affect any permanent tooth in the cervical region, is invasive in nature, and results in progressive destruction of tooth structure. Fibrovascular tissue from the periodontal ligament infiltrates the cervical part of the root during this pathological process [1]. Cementum, enamel, and dentin may gradually resorb, eventually reaching the pulp in more advanced stages [2].

Although the precise cause of ICR is still unknown, several risk factors have been found. These factors were first reported in 1979 by Harrington and Natkin [3]. Orthodontic treatment was the most frequently observed factor (21.2%), followed by trauma (14%). Intracoronal bleaching was a less common isolated factor but was more often present in combination with trauma and/or orthodontic therapy. Bruxism (2%), intracoronal restorations (15.3%), periodontal therapy (4%), and cemento-enamel junction surgery (5.9%) were additional, less common risk factors. There was no discernible risk factor in about 15% of cases [4,5].

Heithersay developed a clinical classification system for ICR, which serves not only as a research tool but also enables comprehensive assessment of treatment outcomes for both surgical and non-surgical approaches. Restoring the defect and deactivating all resorptive tissue should be the main goals of treatment. "Heithersay's approach" included the application of 90% trichloroacetic acid to the resorptive lesion, thereby causing coagulative necrosis, easing the debridement of necrotic tissue. Success rates have been reported to be close to 100% for tiny, circumscribed lesions (class 1 or 2). The success rate for moderately sized lesions (class 3) was 77.8%, but the success rate for severe class 4 lesions was significantly lower at 12.5% [6].

The terminology for ICR has historically been inconsistent, with at least nine different names used in the literature. Heithersay introduced the term “invasive cervical resorption,” which is used in this article [1]. The condition is also sometimes referred to as “extracanal invasive resorption,” as described by Frank and Backland in 1987 [7].

The case presented here can be classified as a class III category based on clinical and radiological examination. Although periapical radiographs were used for diagnosis in this case, recent studies have shown that cone-beam computed tomography (CBCT) offers higher accuracy and sensitivity for detecting and staging external cervical resorption lesions compared with conventional two-dimensional imaging [8,9].

## Case presentation

**Figure 1:**
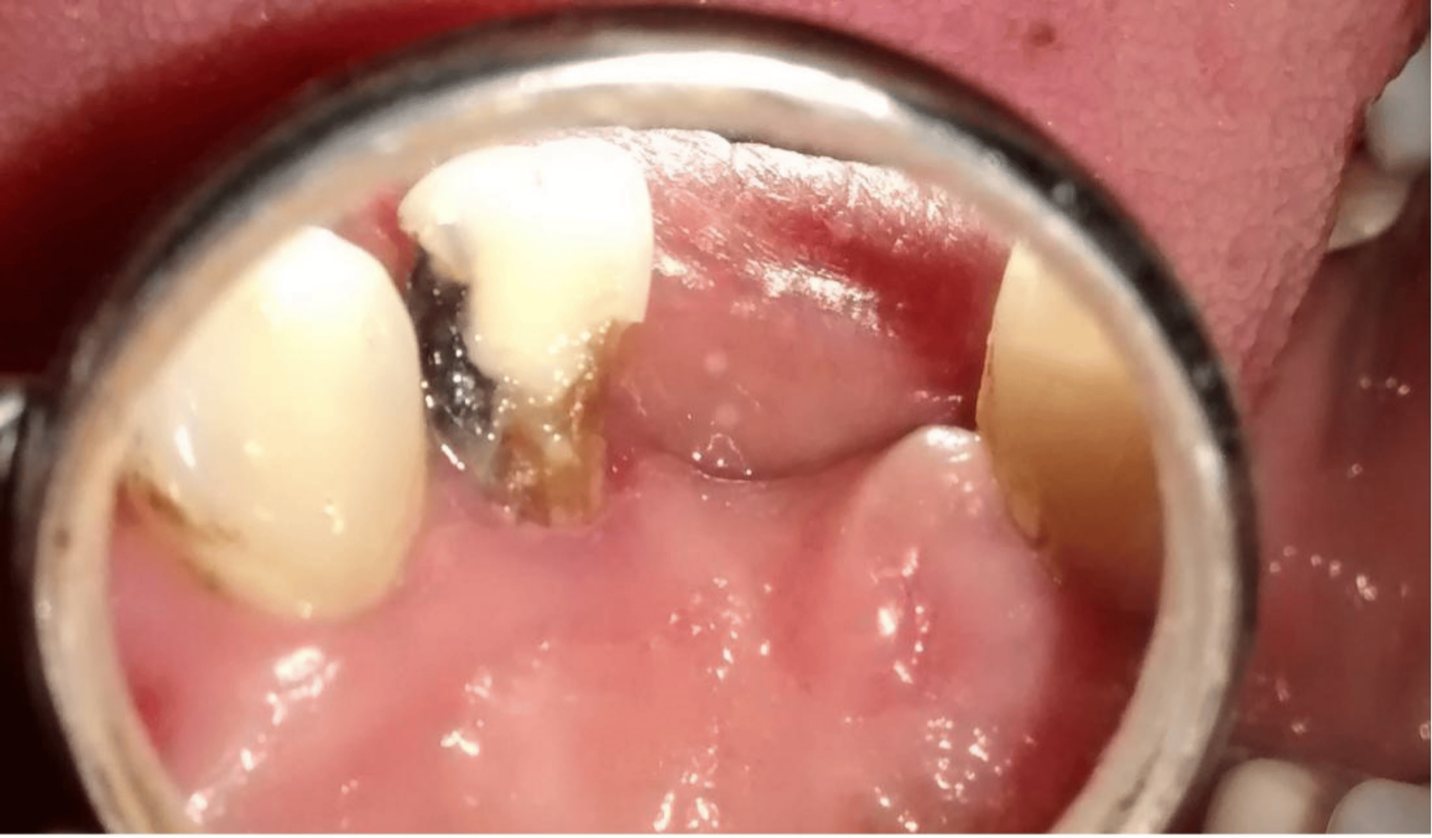
Pre-operative image of the lesion

**Figure 2:**
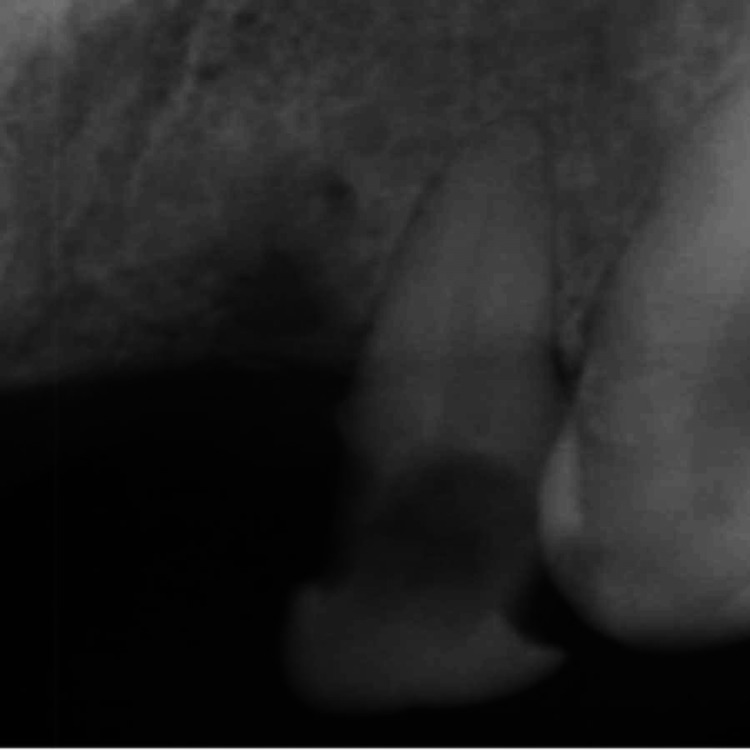
Pre-operative intra-oral periapical radiograph

A 45-year-old female patient presented to the Department of Restorative Dentistry with the chief complaint of a dislodged filling in the upper left front tooth region. She had a history of hard blunt trauma to the front teeth region from a kitchen utensil. She then went to a dentist and was informed about the decay on her tooth. This resulted in a filling being done, which got dislodged later. Clinical evaluation of # 10 revealed a class III recurrent carious lesion and a dislodged restoration on the disto-lingual aspect of the crown. A red-colored defect was noted on the cervical aspect of the crown that did not extend beyond the cemento-enamel junction (Figure 1). Hard tissue was detected on probing the defect, which was also accompanied by a scraping sound. The patient did not report any symptoms. The tooth had a negative response to pulp vitality testing. Periodontal examination revealed normal pocket depths and no mobility. Radiographic examination was consistent with a “moth-eaten appearance” with periradicular anatomy within normal limits (Figure 2).

Management

**Figure 3:**
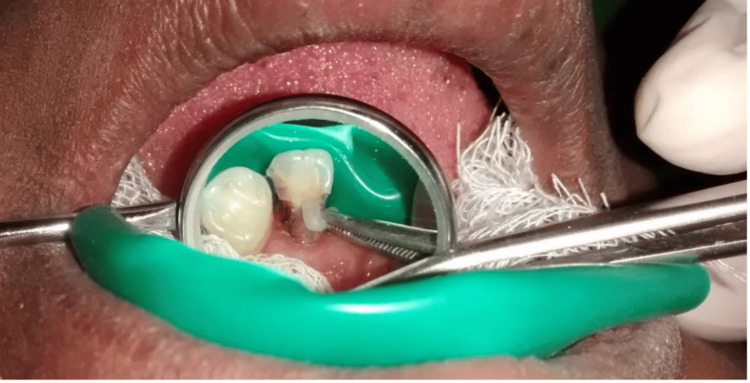
Application of trichloroacetic acid

**Figure 4:**
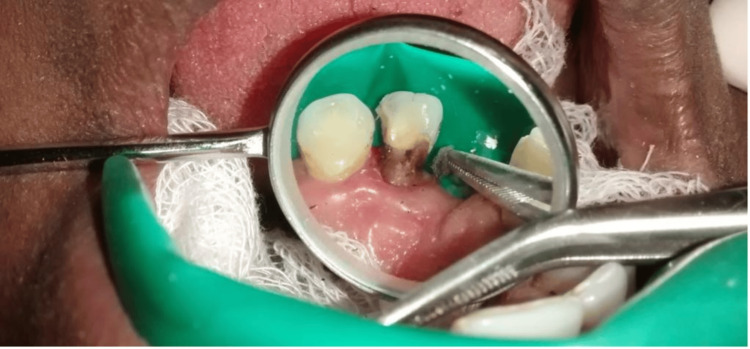
Appearance of coagulative necrosis on the lesion

The Heithersay approach was selected as the treatment of choice owing to the class III category lesion [10]. The maxillary left lateral incisor was isolated using a rubber dam. Trichloroacetic acid (TCA) 90% was applied with a cotton tip applicator to the lesion on the palatal aspect of the tooth for 3-4 minutes (Figure 3). The TCA was continuously applied at least twice until coagulative necrosis of the resorptive tissue was achieved (Figure 4). The necrotic tissue was subsequently removed by curettage using a spoon excavator.

Root canal treatment (RCT) was performed using hand instruments (ICR 10 K-files, Mani, Inc., Japan), with irrigation using 2.5% sodium hypochlorite (Prevest Denpro Limited, Jammu, India), normal saline (Preet International Pvt. Ltd., New Delhi, India), and EDTA 17% (Dentsply Maillefer, Switzerland). The root canal was obturated with zinc oxide eugenol sealer (Vishal Dentocare, Ahmedabad, India) and gutta-percha points (Dentsply) using the lateral condensation technique.

**Figure 5:**
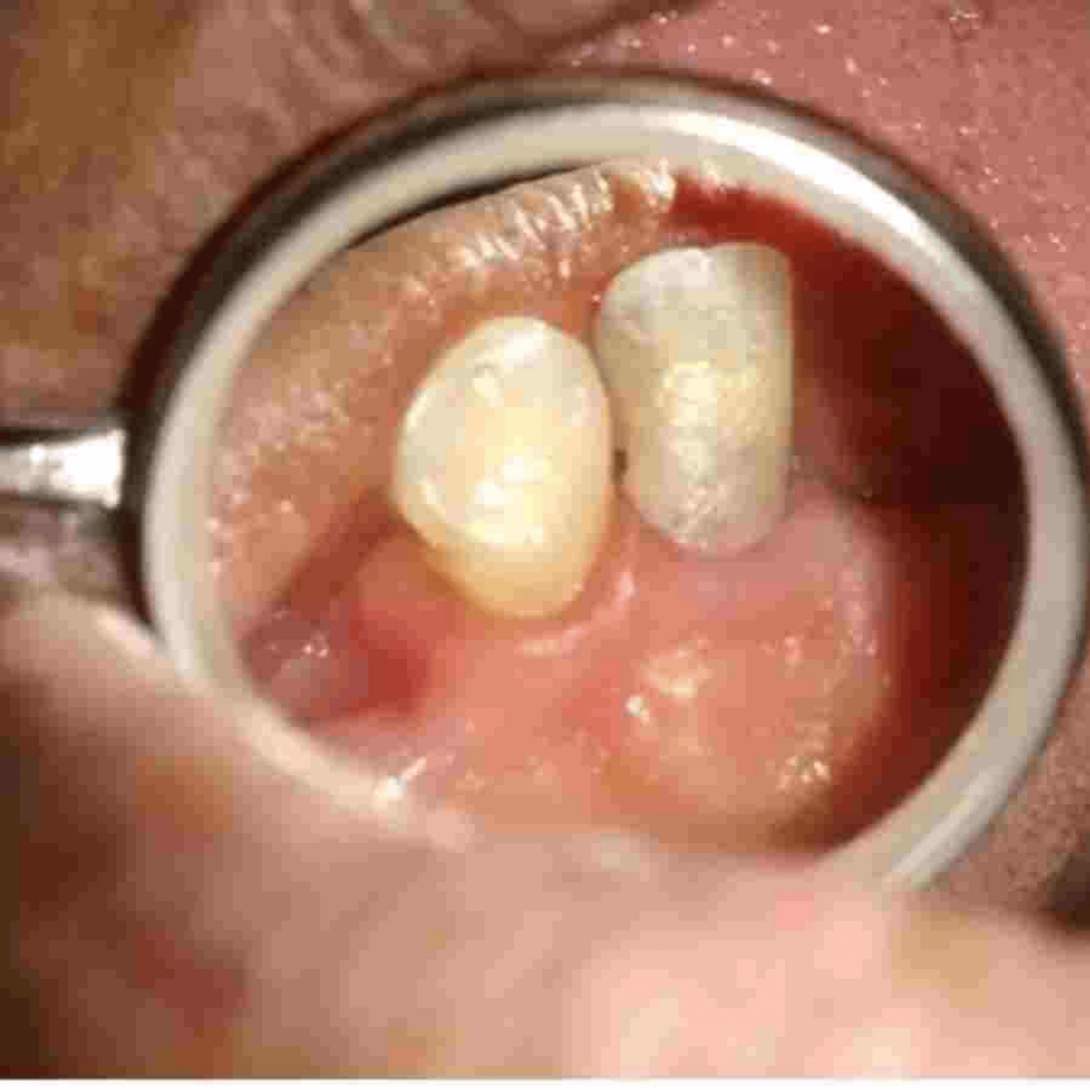
Postoperative image of the tooth

**Figure 6:**
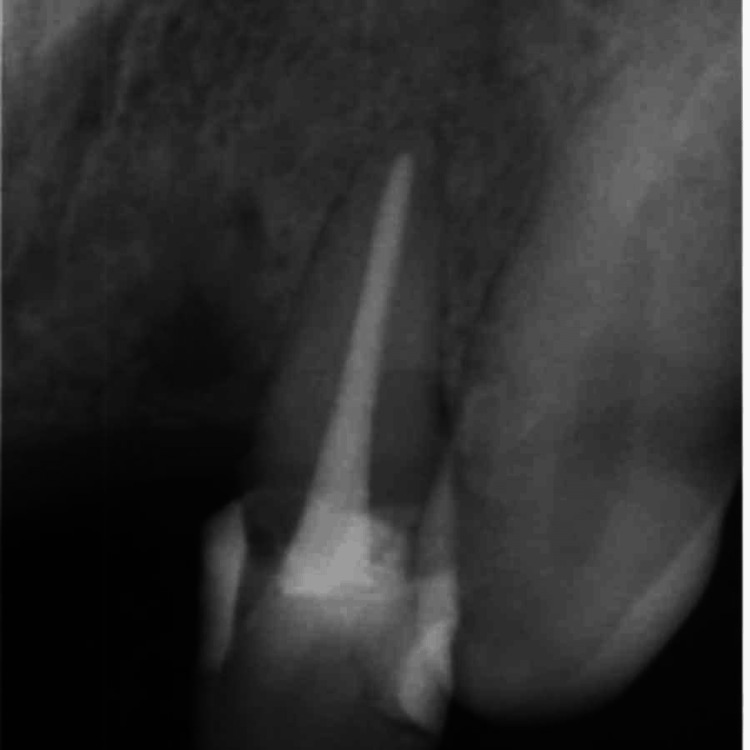
Postoperative intra-oral periapical radiograph

Glass ionomer cement was used to repair the resorptive defect after canal obturation (Figure 5). Radiographs taken after surgery verified that the defect had been satisfactorily restored (Figure 6). Radiographic assessment at the one-year follow-up revealed stable treatment results and no signs of further resorption.

## Discussion

Irrespective of the treatment approach, the main goal in the management of ICR is the complete removal of invasive fibrovascular tissue and a biomimetic restoration of the defect [1]. The restorative material chosen should be esthetically acceptable, biocompatible with the oral environment, and be able to reinforce the already fragile tooth structure [6]. The contributing factors in this case report were trauma and intracoronal restoration. The Heithersay approach was chosen owing to the class III nature of the lesion [10].

The primary aspect of the Heithersay technique is to induce coagulative necrosis of the fibrovascular tissue through the topical application of TCA without harming the adjacent periodontal tissues [2,10,11]. Heithersay has published multiple articles since 1977 that clearly mention the predisposing factors, various clinical features, and the treatment protocols for ICR. The articles especially mention treating class III defects using this method [1,6].

The advantages of this technique are localized eradication of invasive fibrovascular tissue, efficient hemostasis, and avoiding a flap elevation or bone removal, thereby improving patient comfort [12].

TCA selectively targets the invasive cells on the periodontal ligament adjacent to the lesion, thereby helping in better healing [6]. The method of action of TCA is by coagulation of the invasive tissue and the fluids, thereby producing a dry field, which facilitates the placement of the final restorative material. Glass ionomer cement (GIC) was the material of choice in this specific case due to its fluoride-releasing anticariogenic properties, along with rendering strength [11]. TCA, being mildly acidic, also acts as a cavity conditioner prior to the placement of GIC, adequately preparing the enamel and dentin surfaces [13].

Early diagnosis is of paramount importance in the successful management of ICR to ensure the best long-term prognosis.

## Conclusions

Invasive cervical resorption, if not detected in the early stages, may prove detrimental to the overall prognosis, leading to tooth loss. The complexity involved in restoring the missing tooth structure after debridement is also an important factor to be considered. Therefore, early diagnosis is of paramount importance in the successful management of ICR to ensure the best long-term prognosis.
